# Analysis of the Efficiency of Antioxidants in Inhibiting Lipid Oxidation in Terms of Characteristic Kinetic Parameters

**DOI:** 10.3390/antiox13050593

**Published:** 2024-05-11

**Authors:** Sonia Losada-Barreiro, Fátima Paiva-Martins, Carlos Bravo-Díaz

**Affiliations:** 1 Departamento de Química-Física, Facultad de Química, Universidade de Vigo, 36310 Vigo, Spain; sonia@uvigo.es; 2 REQUIMTE-LAQV, Departamento de Química e Bioquímica, Faculdade de Ciências, Universidade do Porto, 4169-007 Porto, Portugal; mpmartin@fc.up.pt

**Keywords:** lipid oxidation, initiation rate, antioxidants, emulsions, antioxidant distribution

## Abstract

In this work, we aim to find physical evidence demonstrating the crucial role that the effective concentration of antioxidants (AOs) present at the interfacial region of emulsions has in controlling the inhibition of the lipid oxidation reaction. We prepared a series of antioxidants of different hydrophobicities derived from chlorogenic and protocatechuic acids. We first monitored, in intact emulsions, the (sigmoidal) production of conjugated dienes and determined the corresponding induction times, *t*_ind_. Independently, we determined the effective concentrations of the antioxidants in the same intact emulsions. Results show that both the length of the induction periods and the antioxidant interfacial concentrations parallel each other, with a maximum at the octyl-dodecyl derivatives. The ratio between the interfacial antioxidant concentrations and the induction periods remains constant for all AOs in the same series, so that the rates of initiation of lipid oxidation are the same regardless of the hydrophobicity of the antioxidant employed. The constancy in the rate of initiation provides strong experimental evidence for a direct relationship between interfacial concentrations and antioxidant efficiencies. Results suggest new possibilities to investigate lipid peroxidation under non-forced conditions and are of interest to formulators interested in preparing emulsions with antimicrobial properties.

## 1. Introduction

Lipid oxidation is an important problem for the food and pharmaceutical industries because it may result in nutrient loss and the potential generation of toxic compounds that may produce “off” odors [1,2,3,4,5,6,7]. Current on-going research is partially focused on developing natural and safe antioxidants that can effectively inhibit lipid oxidation, boosting product stability and shelf-life [4,8,9,10,11].

In this work, we seek to find physical evidence, based on characteristic kinetic parameters, supporting the idea that the efficiency of antioxidants is controlled by their effective concentrations in the interfacial region. For this purpose, we prepared two sets of homologous antioxidants bearing the same reactive moieties but of different hydrophobicity (chlorogenic acid (CGA) and protocatechuic (3,4 dihydroxybenzoic, PCA) acid derivatives) and undertook kinetic studies in attempting to find the relationships between the rate of initiation of the peroxidation reaction, the lengths of the induction periods, and the effective concentrations of antioxidants in the interfacial region of lipid-based oil-in-water emulsions.

We determined the effective concentrations of the antioxidants in intact emulsions (to avoid disruption of existing equilibria) by employing a well-established kinetic methodology in conjunction with the pseudophase kinetic model. The induction periods were determined from kinetic oxidation profiles obtained by monitoring the formation of conjugated dienes, CDs, at the early stage of lipid oxidation with time under conditions in which hydroperoxides undergo little or no decomposition [12]. Although antioxidants may show a different capacity for avoiding the breakdown of hydroperoxides, oxidation starts with the formation of hydroperoxides and not with their breakdown. Their degradation only happens in a later stage of autoxidation, when samples are already oxidized. We have chosen this method because it is a trustworthy and reproducible method to monitor the lipid oxidation reaction in the same intact emulsions as shown by a recent international study [13] and because we demonstrated in previous works that the results obtained by monitoring the formation of CDs are the same as those obtained by monitoring secondary oxidation products [14].

We have chosen CGA and PCA because they are important phenolics with various antioxidant and health benefits [15,16,17,18]. CGA and PCA are commonly present in the daily diet, and their role in active food packing, food control quality, and nutritional dietary supplements is being explored [18,19,20].

## 2. A Brief Overview of the Lipid Oxidation Reaction and Its Inhibition by Antioxidants

**Figure 1:**
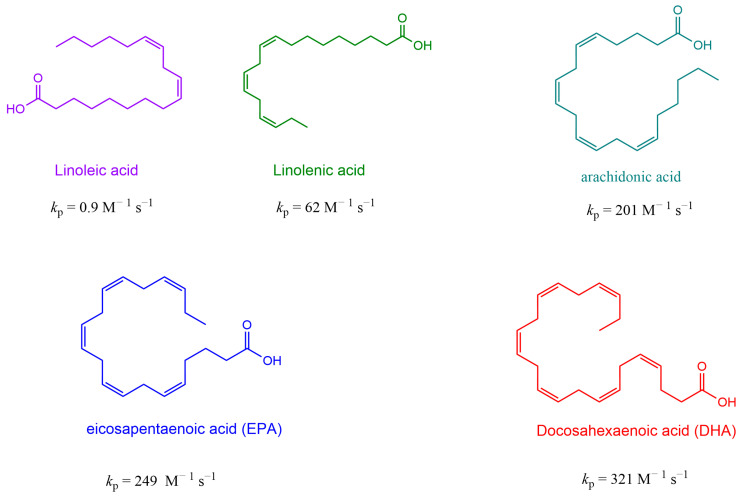
Common unsaturated fatty acids found in animal and vegetable food-grade oils and rate constants *k*_p_ for the propagation step. Values of *k*_p_ are taken from Pratt et al. [10].

Unsaturated lipids are especially prone to autoxidation; the higher the oxidation degree, the easier their oxidation is, as shown in Figure 1 for some representative fatty acids [6,10,21,22].

The mechanism of lipid oxidation in bulk oil is rather complex [23,24], but can be kinetically simplified, bearing in mind the relative rates of the various reactions involved, so that it can be described in terms of the initiation, propagation, and termination steps (Equations (1)–(4)) [1,6,25,26]. The rate-controlling step of the overall oxidation process is the formation of hydroperoxides (Equation (3)) from the reaction of initiating radicals (Equation (1)) with the unsaturated lipids, yielding the corresponding peroxide (Equation (2)) [27,28]. Once hydroperoxides are formed, several reactions may occur, but as a final simplification, two peroxyl radicals can react with each other to form non-radical products through the termination step, Equation (4).

(1) $$\mathrm{Initiation}\;r_{i}\;\mathrm{LH}+\mathrm{In}\;\overset{k_{\mathrm{ini}}}{\to }{\;\mathrm{In}\text{-}H+L}^{•}$$

(2) $${\mathrm{Propagation}\;L}^{•}{+O}_{2}\underset{fast}{\to }{\;\mathrm{LOO}}^{•}$$

(3) $$r_{p}{\;\mathrm{LH}+\mathrm{LOO}}^{•}\overset{k_{p}}{\underset{slow}{\to }}{\;\mathrm{LOOH}+L}^{•}$$

(4) $$\mathrm{Termination}\;r_{t}{\;\mathrm{LOO}}^{•}+{\;\mathrm{LOO}}^{•}\overset{k_{t}}{\to }\;\mathrm{Non}\text{-}\mathrm{radical}\;\mathrm{products}$$

(5) $$\mathrm{Inhibition}\;r_{\mathrm{inh}}{\;\mathrm{ArO}\text{-}H+\mathrm{LOO}}^{•}\overset{k_{\mathrm{inh}}}{\to }{\;\mathrm{ArO}}^{•}+\mathrm{LOOH}\;$$

(6) $${\mathrm{ArO}}^{•}{+\mathrm{LOO}}^{•}\overset{k_{i}}{\to }\;\mathrm{non}\text{-}\mathrm{radical}\;\mathrm{products}$$

During the initiation step, Equation (1), a hydrogen atom next to, or between, double bonds of the unstaturated lipid LH is abstracted, leading to the formation of an alkyl radical L^•^ [21,29]. During the propagation step, these high reactive radicals react with oxygen at diffusion-controlled rates (*k* ≈ 10^9^ M^−1^ s^−1^), leading to the formation of peroxyl radicals LOO^•^, Equation (2). In turn, these radicals may react with unsaturated fatty acids LH to produce either lipid peroxides LOOH according to Equation (3) (the first metastable oxidation products) or eventually new alkyl radicals [25,30]. These reactions are rate-determining, and they are usually known as the propagation step because each reaction generates another radical, sustaining the propagation cycle and keeping the radical chain alive [10,21].

The consumption of oxygen is given by Equation (7) where *k*_p_ is the rate constant for the propagation reaction (Equation (3)):

(7) $$-\;\frac{{d[O}_{2}]}{\mathrm{dt}}=k_{p}{[\mathrm{LOO}}^{•}][\mathrm{LH}]$$

In bulk solution, the elementary steps indicated by Equations (2)–(5) take place concurrently, each one having its own rate constant. Denoting as *r*_i_ the rate of the initiation step (Equation (1)), and assuming that the steady-state condition holds (i.e., rate of chain initiation = rate of termination), the general equation for the uninhibited oxygen uptake is given by Equation (8) where *k*_t_ is the rate constant for the termination reaction (Equation (4)) [31]. The ratio *k*_p_/(2*k*_t_)^1/2^ is the oxidizability index and mainly depends on the degree of unsaturation of the fatty acids. For example, extra olive virgin oil with an 80% oleic acid content has a much lower oxidizability index than sunflower oils, which typically contain only 20% oleic acid but 60% linoleic acid [31].

(8) $$-\;\frac{{d[O}_{2}]}{\mathrm{dt}}=\frac{k_{p}}{{\left(2k_{t}\right)}^{1/2}}r_{i}^{1/2}\left[\mathrm{LH}\right]$$

For accurate kinetic studies, it is necessary to control the production of initiating radicals, Equation (1) [10,27,28,31]. Such a control can be achieved by using azo-dye initiators with well-known decomposition rates or by keeping samples under identical experimental conditions (as we performed in the present study to avoid the addition of unnecessary chemicals). The latter approach is especially useful when comparing the effects of added antioxidants on the rate of lipid oxidation. Such controlled conditions facilitate the assessment of the antioxidant efficiency in inhibiting peroxidation, particularly when considering relationships between the structure of the antioxidants, their effective concentration in the various regions of the emulsions, and the propagation reaction [28,32,33].

The competition between antioxidants and lipids for peroxyl (chain-carrying) radicals means that their reactivity can be readily determined based on the progress of the inhibited peroxidation reaction. Efficient antioxidants are those who have: (1) high reactivity (i.e., high inhibition rate constant *k*_inh_) values such that *k*_inh_ >> *k*_p_; (2) the effective concentration of the antioxidant in the interfacial region is high enough to ensure that r_inh_/r_p_ >> 1. In addition to these properties, it is also desirable that the efficient antioxidant must also be chemically stable and should not behave as a chain-transfer agent, that is, the product(s) derived from its reaction with the peroxyl radicals should not initiate new chain reactions nor contribute to carrying them on [1,10]. Since peroxyl radicals propagate peroxidation, most commonly by H-atom transfer (HAT) [10], it would be desirable to use antioxidants with a relatively weak ArO-H bond, and the bond dissociation energy generally correlates with *k*_inh_ values [10,21].

Literature reports on the kinetics and mechanism of inhibition reactions in micellar systems demonstrate that the kinetics of peroxidation follow the same rate laws as for homogeneous solutions but require considering the corresponding rate constants at the various reaction sites and the partitioning of reactants between regions [34]. Therefore, a thorough description of the oxidation reaction requires taking into account the partitioning of the reactants between the various domains of the colloidal system [27,35,36,37].

### 2.1. Addition of Chain-Breaking Antioxidants: Kinetic Effects

Before presenting the experimental results, it is worthwhile to analyze the kinetic effects of the addition of the chain-breaking antioxidant ArOH to emulsions where unsaturated lipids are present. When ArOH is added to inhibit peroxidation, most of the peroxyl radicals are trapped by the antioxidant, so a new steady-state condition applies. The rate-limiting step is the reaction between the antioxidant ArOH and the peroxyl radical, given by Equation (5), which leads to much fewer reactive radicals derived from the antioxidant. The recombination reaction between ArO^•^ and LOO^•^, Equation (6), is fast and is the origin of the stoichiometric factor n, the number of peroxyl radicals trapped by the antioxidant in reaction 6, with a typical value of n ≈ 2 for phenolic antioxidants [31].

The relationship between the rate of the inhibited peroxidation and the rate of initiation is given by Equation (9), where the ratio *k*_p_/k_inh_ stands for the antioxidant efficiency AE [28,31,34], reflecting the competition between the reactions given by Equations (3) and (5). The higher the AE is, the higher the efficiency of the antioxidant in inhibiting the lipid peroxidation reaction.

(9) $$-\;\frac{{d[O}_{2}]}{\mathrm{dt}}=\frac{k_{p}}{k_{\mathrm{inh}}}\left[\mathrm{LH}\right]\frac{r_{i}}{n\left[\mathrm{ArOH}\right]}$$

Equation (9) provides a useful and quantitative understanding of the efficiency of antioxidants. In bulk solution, the “ability” of an antioxidant to suppress the lipid oxidation reaction depends on the value of the ratio *k*_p_/*k*_inh_, that is, for an antioxidant to be effective in bulk solution, the inhibition rate constant must be much higher than that for the propagation step [28]. By definition, the inhibited reaction takes place in the presence of an antioxidant until most of the antioxidant is consumed, and then the oxidation reaction returns to its uninhibited rate, so that Equation (3) applies [28,29,38].

**Figure 2:**
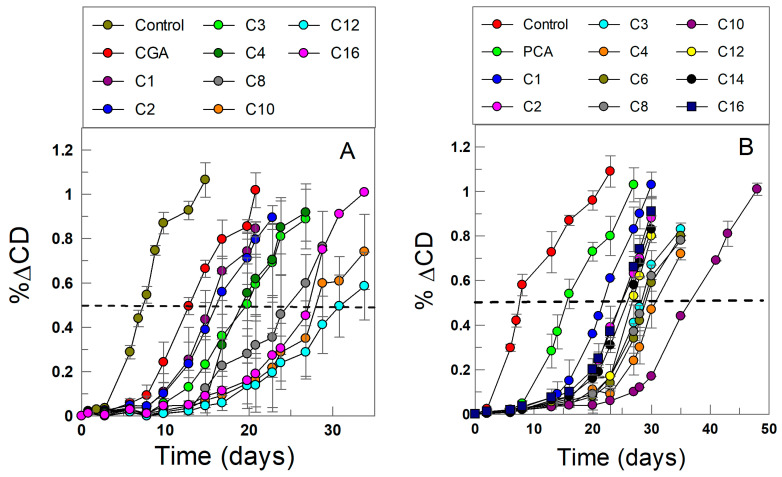
Kinetics of lipid oxidation in the absence (control) and in the presence of CGA (**A**) and PCA (**B**) antioxidant derivatives in emulsions. The dashed line at 0.5% ΔCD (increase in the percentage of conjugated dienes, CDs) was employed to assess the relative efficiency of the antioxidants [13], and matches that determined by measuring the length of the induction period (see results section). Experimental conditions: olive oil 4:6 (*o*/*w*, *v*:*v*) emulsions prepared with citric acid/citrate buffer (0.04 M, pH = 3.65), surfactant volume fraction Φ_I_ = V_surf_/V_emulsion_ = 0.01, [AO_T_] = 2.4 × 10^−4^ M, T = 60 °C.

Figure 2 shows the typical profile for a lipid oxidation reaction in the presence of antioxidants, showing the slow formation of peroxidation products and the characteristic induction period. In bulk solution, the rate of chain initiation can be calculated by employing Equation (10), where *τ*_ind_ is the length of the induction period, n is the stoichiometric factor, and [ArOH] is the stoichiometric concentration of the antioxidant [27,28,31,34].

(10) $$r_{i}=\frac{n[\mathrm{ArOH}]}{\tau_{\mathrm{ind}}}$$

### 2.2. Inhibited Lipid Oxidation Reactions in Emulsions: Highlighting the Critical Role of the Effective Concentrations of Antioxidants in the Oil, Aqueous, and Interfacial Regions

In lipid dispersions, reactants move from one lipid droplet to another and within the various (oil, water, and interfacial) regions of the emulsion [39,40,41]. Lipid droplets breakdown and reform at rates that are much higher than the rate-limiting reactions described above, and hence, from the point of view of the reaction, lipid droplets behave as “static” structures no matter their size; that is, the reaction “sees” the emulsion droplets as static entities. This means that lipid oxidation in aqueous lipid dispersions is primarily an interfacial phenomenon where the interface, which is the three-dimensional region between the oil core and bulk aqueous solution, plays a critical role and where the concentration of the various reactive species is crucial to the fate of both lipid oxidation and its inhibition.

The fact that the reaction “sees” the lipid emulsion droplets as static entities implies that the dynamics of the droplets do not need to be considered in terms of the kinetics of the reactions so that the observed rate of the reaction can be computed as the sum of the rates in all regions of the emulsion [28,33,37,42]. A relatively modest mathematical model, based on the theory of the transition state and that takes into account simple diffusion laws, permitted Bravo et al. [28,43] to analyze the conditions required to distinguish between kinetically controlled and diffusion controlled systems. The analyses were carried out under the assumption that the transport of reactants between the various regions of the emulsion is not restricted by physical barriers (as it happens in the emulsions we have employed here) [28,43].

On these grounds, Bravo et al. [28,43,44] showed that the rates of the propagation and inhibition reactions (Equations (3) and (5)) are much smaller than the diffusion of reactants within and between droplets. So, at any time a reactant undergoes a chemical reaction, it is instantly substituted by another reactive molecule from the same or from a nearby region [28]. Because in emulsified systems the chemical reactions are governed by the same rate laws as in bulk solution, the overall rate of the inhibition reaction is given by the sum of the rates of the inhibition reactions in the oil (O), interfacial (I), and aqueous (W) regions, Equation (11), where the parenthesis indicates the effective concentrations expressed as moles per liter of a particular region.

(11) $$\begin{array}{l} r_{\mathrm{inh}}=r_{\mathrm{inh}(W)}+r_{\mathrm{inh}(I)}+r_{\mathrm{inh}(O)}=\\ =nk_{\mathrm{inh}(W)}{(\mathrm{LOO}}_{W}^{•}{)(\mathrm{ArOH}}_{W})+nk_{\mathrm{inh}(I)}{(\mathrm{LOO}}_{I}^{•}{)(\mathrm{ArOH}}_{I})+nk_{\mathrm{inh}(O)}{(\mathrm{LOO}}_{O}^{•}{)(\mathrm{ArOH}}_{O}) \end{array}$$

The kinetic analyses of the reaction in emulsified systems are tremendously complicated by the partitioning of the reactants because the overall rate of inhibition *r*_inh_ depends now on both the particular values of the rate constant *k*_inh_ in each region and on the real or actual concentrations of reactants at the reaction site [28].

As discussed elsewhere [28,33,44], the values of *k*_inh(w)_ and *k*_inh(O)_ could be obtained eventually from independent measurements, but not those of the interfacial region *k*_inh(I)_ because it is a highly anisotropic region whose exact composition is unknown. Moreover, the effective concentration of antioxidants in each region is unknown, and it is different from the stoichiometric one [28].

To preclude the risk of biasing the experimental results, determining the effective concentrations in emulsions needs to be performed in the intact emulsions [28,37]. Researchers around the world developed creative protocols in attempting to determine the real concentrations of reactants in the various regions. A literature inspection indicates most of the existing methods were based on separation (centrifugation or ultrafiltration) techniques in combination with quantitative analysis of reactants in each separated phase. Stöckmann and Schwarz [45,46,47] used the mentioned techniques in combination with a mathematical model to estimate the distribution of phenol derivatives in emulsions. However, the results were not as satisfactory as desired [45]. An adaptation of the method was employed later by Sorensen et al. [48], assuming that the distribution equilibrium of antioxidants is not significantly disrupted after breaking down the emulsion separating the two phases by centrifugation. However, in both methods, the existing equilibria are modified before the quantitative analyses, and hence none of those methods provide real estimates of interfacial concentrations [47,49,50].

**Figure 3:**
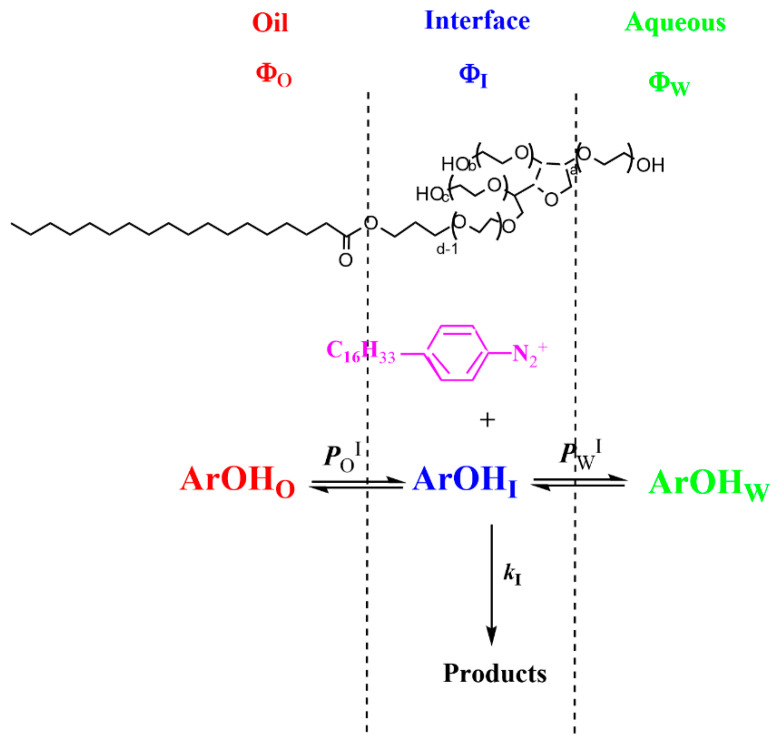
Explanatory representation of an emulsion droplet showing the location of the chemical probe 4-hexadecylbenzenediazonium (16-ArN_2_^+^) in the interfacial region of the droplet, where it reacts with the antioxidant ArOH, which is distributed between the oil (O), interfacial (I), and aqueous (W) regions. Φ stands for the volume fraction of a region, defined as Φ = V_region_/V_emulsion_, and the partition constants between the aqueous-interfacial, *P*_W_^I^, and oil-interfacial, *P*_O_^I^, regions are defined by Equations (12) and (13), respectively.

The use of chemical probes in combination with physical-organic chemistry methods, grounded in thermodynamics and inspired by pseudophase models [28,37,51], was proposed by Romsted et al. [52] as an alternative, useful, non-destructive methodology. The method exploits the use of suitable chemical probes located in the interfacial region of the emulsions, to assess the distribution of antioxidants in the intact emulsions [28,37]. Briefly, the probe reacts with the antioxidants in the interfacial region of the emulsion, as illustrated in Figure 3, and mathematical relationships between the observed rate constants (*k*_obs_) and the emulsifier fraction (Φ_I_) were derived, from which the values of the partition constants were estimated [28].

### 2.3. Overview of the Pseudophase Kinetic Model to Determine the Distribution of AOs between the Oil-Interfacial and Aqueous-Interfacial Regions of the Emulsions

The chemical kinetic method exploits the overall bimolecular reaction between hydrophobic 4-hexadecylbenzenediazonium, 16-ArN_2_^+^ ions, and an AO (ArOH in the Figure 3). 16-ArN_2_^+^ is located completely in the interfacial region, but the distributions of the AOs depend on their relative solubilities in the oil, interfacial, and aqueous regions, Figure 3. Consequently, two partition constants, *P*_W_^I^ and *P*_O_^I^, are now necessary to describe the distribution of the antioxidant between the aqueous and interfacial regions *P*_W_^I^ and between the oil and interfacial regions *P*_O_^I^, Equations (12) and (13), respectively [28].

(12) $$P_{W}^{I}=\frac{{(\mathrm{AO}}_{I})}{{(\mathrm{AO}}_{W})}$$

(13) $$P_{O}^{I}=\frac{{(\mathrm{AO}}_{I})}{{(\mathrm{AO}}_{O})}$$

Because the reaction occurs only in the interfacial region, the interfacial and total concentrations of 16-ArN_2_^+^ are equal, and the observed rate, v, and *k*_obs_ are directly proportional to the concentration of the antioxidant in the interfacial region, Equation (14).

(14) $$Observed\;Rate=Rate\;in\;Interfacial\;Region=k_{I}\cdot \left(16-Ar{N_{2}^{+}}_{I}\right)\cdot \left(AO_{I}\right)\cdot \Phi_{I}$$

where subscript I stands for the interfacial region, *k*_I_ is the second-order rate constant in the interfacial region, parentheses () indicate concentration in moles per liter of the volume of a particular region, and Φ_I_ is the surfactant volume fraction, defined as Φ_I_ = V_surf_/V_total_, which is assumed to be equal to that of the interfacial region.

Equation (15) can be derived in terms of measurable parameters by combining Equation (14) with the mass balance equations for AO and 16-ArN_2_^+^ and the definitions of the partition constants, *P*_W_^I^ = (AO_I_)/(AO_w_) and *P*_O_^I^ = (AO_I_)/(AO_O_). Equation (15) describes the dependence of *k*_obs_ on both the AO concentration (*P*_W_^I^ and *P*_O_^I^) and medium effects (*k*_I_).

(15) $$k_{\mathrm{obs}}=k_{I}\left({\mathrm{AO}}_{I}\right)=\frac{\left[{\mathrm{AO}}_{T}\right]k_{I}P_{W}^{I}P_{O}^{I}}{\Phi_{O}P_{W}^{I}+\Phi_{I}P_{W}^{I}P_{O}^{I}+\Phi_{W}P_{O}^{I}}$$

The partition constants are determined by fitting the experimental data to Equation (15) in combination with Equation (16), as described elsewhere [28].

(16) $$P_{W}^{O}=\frac{P_{W}^{I}}{P_{O}^{I}}=\frac{\left({\mathrm{AO}}_{O}\right)}{\left({\mathrm{AO}}_{W}\right)}$$

Depending on the hydrophobicity of the antioxidant, Equation (15) simplifies: (i) when the reactant (AO) is aqueous insoluble (i.e., very hydrophobic), only *P*_O_^I^ is needed to describe a reactant distribution, and the relationship between *k*_obs_ and Φ_I_ is given by Equation (17); (ii) when the reactant is oil insoluble (i.e., very hydrophilic), only the partition constant *P*_W_^I^ is needed to describe the distribution of a reactant, and the relationship between the *k*_obs_ and Φ_I_ is given by Equation (18). Once the partition constants are known, determining the percentage of the antioxidant in the interfacial region of the emulsion is straightforward.

(17) $$k_{obs}=\frac{k_{I}{\left[\mathrm{AO}\right]}_{T}P_{O}^{I}}{\Phi_{I}P_{O}^{I}+\Phi_{O}}$$

(18) $$k_{obs}=\frac{k_{I}{\left[\mathrm{AO}\right]}_{T}P_{W}^{I}}{\Phi_{I}P_{W}^{I}+\Phi_{W}}$$

The percentage of the AO in the interfacial region is obtained by using Equations (19)–(21) and the calculated values of *P*_W_^I^ and *P*_O_^I^. Details are given elsewhere [28,37].

(19) $${\%\mathrm{AO}}_{I}=\frac{100\Phi_{I}P_{O}^{I}P_{W}^{I}}{\Phi_{O}P_{W}^{I}+\Phi_{I}P_{O}^{I}P_{W}^{I}+\Phi_{W}P_{O}^{I}}$$

(20) $${\%\mathrm{AO}}_{I}=\frac{100\Phi_{I}P_{W}^{I}}{\Phi_{I}P_{W}^{I}+\Phi_{W}}$$

(21) $$\%{\mathrm{AO}}_{I}=\frac{100\Phi_{I}P_{O}^{I}}{\Phi_{I}P_{O}^{I}+\Phi_{O}}$$

## 3. Materials and Methods

### 3.1. Chemicals and Materials

All chemicals and solvents were of the highest purity available from Sigma-Aldrich (Darmstadt, Germany) and/or Acros Organics (Geel, Belgium) and were used as received. Aqueous solutions were prepared with deionized water (conductivity < 0.1 mS cm^−1^). Buffered aqueous solutions were prepared by employing citric acid/citrate buffer (0.04 M, pH 3.65), and their pH was measured by potentiometry.

The chemical probe 4-hexadecylbenzenediazonium, 16-ArN_2_^+^ (prepared as tetrafluoroborate), was employed to evaluate the distribution of the antioxidants in the emulsions. It was prepared in high yield and purity from commercial 4-hexadecylaniline (Sigma-Aldrich) by diazotization [53] and stored in the dark at a low temperature to minimize its spontaneous decomposition. The coupling agent N-(1-Naphthyl)ethylenediamine (NED) solution, employed to monitor the reaction between the chemical probe 16-ArN_2_^+^ and the antioxidants, was prepared in a 50:50 (*v*:*v*) BuOH:EtOH mixture to give a final [NED] = 0.02 M.

Extra virgin olive oil was purchased in a local store and stripped of its endogenous antioxidants by following literature procedures, washing it with a 0.5 M NaOH solution, and passing twice through a previously activated aluminum oxide column. The complete removal of endogenous antioxidants was confirmed by HPLC according to the IUPAC method 2.432. Details can be found elsewhere [54]. The stripped oil was kept at a low temperature in an inert atmosphere and in the dark to minimize its spontaneous peroxidation.

The surfactant Tween 20, employed to prepare the emulsions, protocatechuic and chlorogenic acids, and the alcohols employed in the preparation of their hydrophobic esters were purchased from Acros Organics (Geel, Belgium) and used as received. Thin layer chromatography (TLC) analyses were performed on aluminium silica gel sheets 60 F_254_ plates (Merck, Darmstadt, Germany), and spots were detected using a UV lamp at 254 nm and iodine.

### 3.2. Synthesis of Fatty Acid Esters

**Figure 4:**
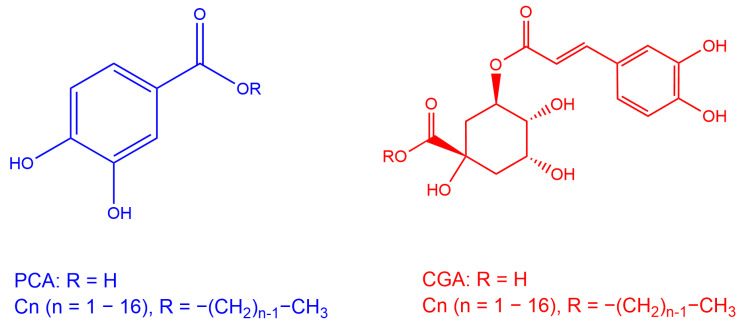
Chemical structures of the protocatechuic (PCA) and chlorogenic (CGA) acid derivatives (C1–C16) employed in this work. The antioxidants were chosen because they are natural catecholic compounds with interesting properties related to health [56,57,58].

Chlorogenate and protocatechuate esters (C_2_–C_4_) were synthesized by chemical acylation of the carboxylic group or by enzymatic acylation (C_6_–C_16_ derivatives) following the procedures described elsewhere [55]. The chemical structures of the synthesized antioxidants are shown in Figure 4.

Briefly, the (C2–C4) esters were prepared by dissolving the parent acid (CGA 1.4 mmol, PCA 6.5 mmol) in the desired fatty alcohol. 1–2 mL of the catalyst (H_2_SO_4_ 97%) were added to the reaction mixture, which was then stirred at room temperature. The solvent was partially evaporated, and the solution was neutralized with aqueous Na_2_CO_3_ 2M. The final solution was extracted with diethyl ether and dried over Na_2_SO_4_. The solvent was evaporated, and the product was purified by recrystallization with acetone (CGA) or hexane (PCA).

The C6–C16 CGA esters were prepared by adding the parent acid (CGA 1.4 mmol) to a mixture of corresponding fatty alcohol (14.1 mmol) and 2 mL of THF containing Novozym 435 (0.52 g) and molecular sieves (0.180 g) in a dry round bottom flask and stirred for 7 days at T = 65 °C. The esters were purified in a two-step procedure. First, the enzyme and molecular sieves were removed by decanting off the solution, 80 mL of ethyl acetate was added, and the combined solution was extracted with aqueous 0.6 M Na_2_CO_3_ and the solvent evaporated. In a second step, alcohol traces and the esters were removed using flash column chromatography over silica gel using toluene/ethyl acetate (9:1, *v*/*v*) and dichloromethane/methanol (10:0.75, *v*/*v*) as eluents, respectively. The C_6_–C_16_ PCA esters were prepared and purified in a two-step procedure as described elsewhere [59].

Reactions were monitored by thin layer chromatography (TLC) on precoated aluminum silica gel sheets 60 F254 plates (Merck, Darmstadt, Germany), and spots were detected by using a UV lamp at 254 nm and iodine. In all cases, final yields (purified compounds, purity > 98%) were 65–75% for the C_1_–C_4_ derivatives and 50–65% for the C_6_–C_16_ derivatives.

Nuclear magnetic resonance (NMR) spectra were recorded on 400 or 100 MHz NMR equipment with CDCl_3_ as solvent. In all cases, ^1^H and ^13^C NMR spectra of the synthetized antioxidants were in accordance with those reported in the literature [60].

### 3.3. Emulsion Preparation

Emulsions were prepared by employing the stripped oil, acidic water (0.04 M citrate buffer, pH 3.65), and Tween 20 (0.5–4%, *w*/*w*), and the mixtures were stirred at high speed at room temperature with the aid of a Polytronic PT-1600 homogenizer, as in previous works of our group [28].

### 3.4. Monitoring the Formation of Primary Oxidation Products

The relative antioxidant efficiency in emulsions was determined, as in previous works, by monitoring the formation of primary oxidation products (conjugated dienes, CDs) with time [28]. The procedure is reliable and trustworthy as recently demonstrated in an international, inter-laboratory, study [13]. Emulsions prepared in the absence and in the presence of a fixed concentration of AO were placed in a thermostated orbital shaker (T = 60 °C, 500 r.p.m.) in the dark, and they were allowed to spontaneously oxidize. At selected times, 50 µL of each emulsion were diluted to 10 mL with ethanol, and the absorbance at λ = 233 nm was measured, and plots of the variation in the formation of CDs with time were prepared as illustrated in Figure 2. All runs were performed in triplicate to minimize errors.

### 3.5. Determining the Partition Constant, P_W_^O^, in Binary Oil-Water Mixtures

The partition constant *P*_W_^O^ between olive oil and water was determined by employing a shake-flask method as in previous works [14]. 4:6 (*v*:*v*) binary oil/water mixtures containing each AO (final stoichiometric concentration = 3.5 mM) were prepared and thermosted at T = 25 °C. The percentages of AO in each phase were determined by UV-VIS spectrometry, as described elsewhere [28]. *P*_W_^O^ was determined by employing Equation (22) where V_W_ and V_O_ are the volumes of the aqueous and oil phases, respectively. The calculated values are the average of three runs.

(22) $$P_{W}^{O}=\frac{{(\mathrm{AO}}_{O})}{{(\mathrm{AO}}_{W})}=\frac{{\%\mathrm{AO}}_{O}}{{\%\mathrm{AO}}_{W}}\times \frac{V_{W}}{V_{O}}$$

### 3.6. Determination of the Observed Rate Constant, k_obs_, for the Reaction between 16-ArN_2_^+^ and the AOs in Olive Oil Emulsions

A special protocol described in detail elsewhere [37] was used. Reactions between the chemical probe and the antioxidant were carried out under pseudo-first-order conditions, [AO] >>> [16-ArN_2_^+^], and monitored for at least 2–3 *t*_1/2_. *k*_obs_ values were obtained by fitting the absorbance-time pairs of data to the integrated first-order Equation (23), using a non-linear least squares method provided by a commercial computer program (GraFit 5.0.5). In Equation (23), A_t_, Ao, and A_inf_ are the measured absorbances at any time, at *t* = 0, and at infinite time.

**Figure 5:**
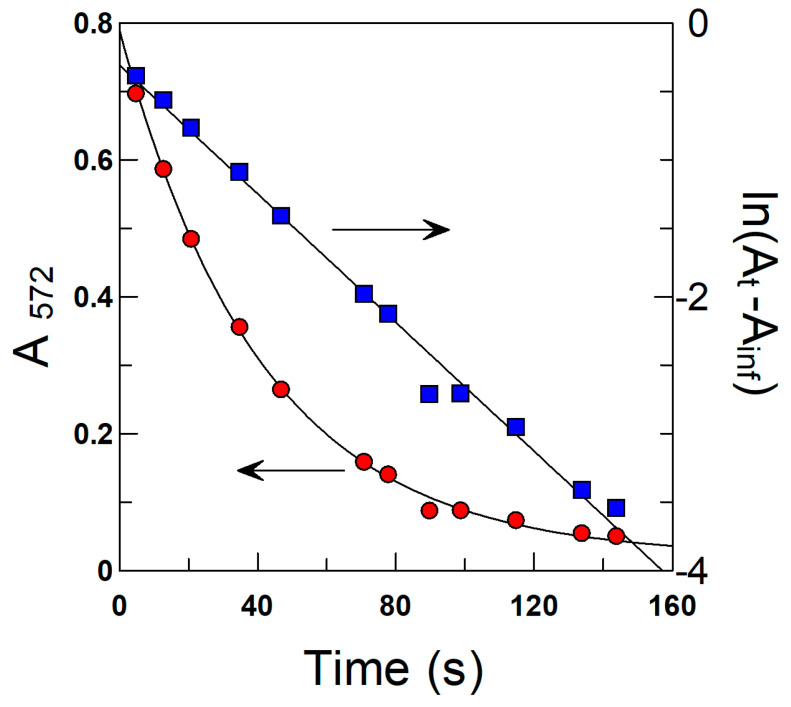
Illustrative example of the determination of the observed rate constant *k*_obs_ for the reaction between butyl chlorogenate (C4-CGA) and the chemical probe 16-ArN_2_^+^ in 4:6 olive oil emulsions (Figure 3). Solid lines are the theoretical curves obtained by fitting the experimental data to the integrated first-order equation (Equation (23)). Experimental conditions: T = 25 °C, pH = 3.65 (citric acid/citrate buffer), Φ_I_ = 0.005, [16-ArN_2_^+^] = 2.81 × 10^−4^ M, [C4-CGA] = 0.003 M.

An illustrative example of the kinetic plots commonly obtained is given in Figure 5 [28]. The correlation coefficients were > 0.99 in all runs. Duplicate or triplicate experiments gave *k*_obs_ values with deviations lower than 7%.

(23) $$\ln\left(A_{t}{-A}_{\inf}\right)={\ln(A}_{0}{-A}_{\inf})-k_{\mathrm{obs}}t$$

### 3.7. Statistical Analysis

Kinetic experiments were run in duplicate or triplicate for 2–3 t_1/2_. The *k*_obs_ values were within ±7–9%, with typical correlation coefficients of >0.995. Oxidation kinetic experiments were run in triplicate. The SPSS 21.0 software was used for one-way analysis of variance (ANOVA, with Tukey’s HSD multiple comparison). The level of significance was set at *p* < 0.05. Data are presented as means ± standard deviation. Acceptance or rejection of the datum (before calculating the average of the set of replicates) was decided based on Dixon’s Q-test.

## 4. Results and Discussion

### 4.1. Oxidative Stability of Olive Oil-in-Water Emulsions: Antioxidant Efficiency

**Figure 6:**
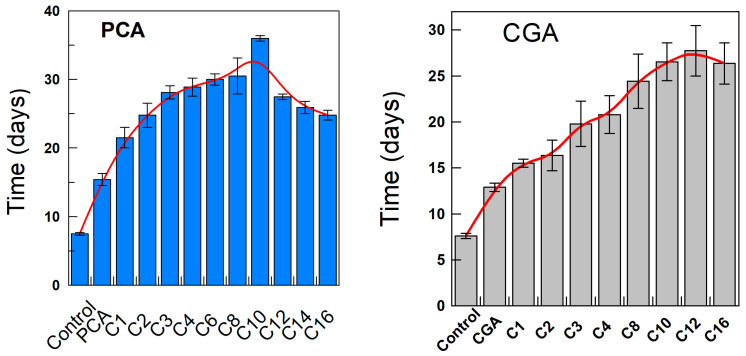
Variation of the time necessary to reach an increase in the percentage of CDs of 0.5% with the hydrophobicity (number of C atoms in the alkyl chain) of PCA and CGA esters.

The relative AO efficiency of PCA and CGA esters in 4:6 (*o*/*w*) olive oil-in-water emulsions was investigated, as in previous works [13,61,62], by measuring the formation of the primary oxidation product, conjugated dienes (CDs). The relative efficiency was determined at T = 60 °C by measuring the time required to increase the CD content by 0.5% both in the absence (control experiment) and in the presence of AOs, measured after the propagation step had been initiated (dashed lines in Figure 2). The variation of the time with the length of the alkyl chain is shown in Figure 6 for PCA (7A) and CGA (7B), and the relative order is in keeping with that obtained by determining the induction times.

As observed, a maximum is obtained for the C6–C8 derivatives (PCA) and for the C10–C16 derivatives (CGA), indicating that their efficiency in inhibiting lipid oxidation does not correlate with the hydrophobicity of the antioxidants. This parabola-like variation of the efficiency with the number of C atoms in the alkyl chain is in keeping with the so-called “cut off” effect previously reported by ourselves [28,63] and others [64,65,66,67] for a series of homologous AOs bearing the same reactive moieties but of different hydrophobicity. Results in Figure 6 are also, qualitatively, similar to those reported by Laguerre et al. in Brij 35 stabilized sunflower oil-in water emulsions [64].

We note that the AOs employed here constitute two sets of AOs bearing the same reactive groups but different alkyl chains. We previously demonstrated that varying the alkyl chain of AOs has a negligible effect on their reactivity against commercial radicals such as DPPH^•^ [14]. Thus, changes in efficiency are likely to be a consequence of changes in their relative concentrations in the oil, interfacial, and water regions, in keeping with previous results [28,52,63]. To further test the hypothesis, we determined the AO distributions and their effective concentrations in the very same intact emulsions as those employed in the oxidation kinetics experiments.

### 4.2. Distribution of Antioxidants between the Oil, Interfacial, and Aqueous Regions of Emulsions

**Figure 7:**
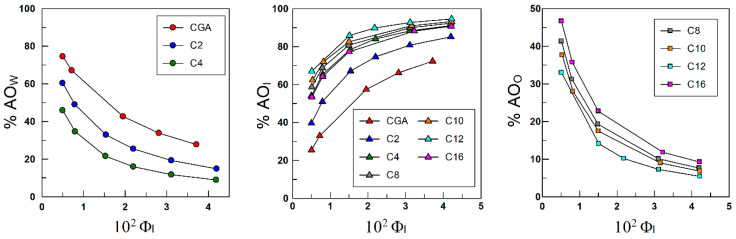

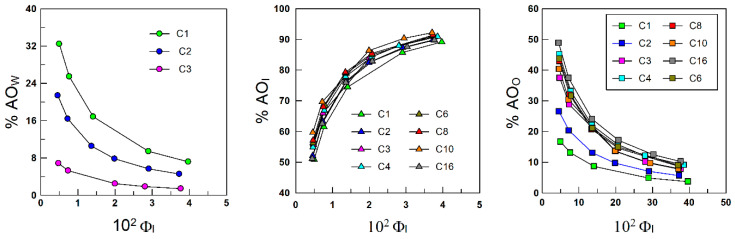
Distribution of CGA (**upper**) and PCA (**lower**) antioxidants between the aqueous (W), interfacial (I), and oil (O) regions of olive 4:6 (*o*/*w*, *v*:*v*) emulsions. As illustrated, antioxidants are transferred to the interfacial region from the aqueous and oil regions upon increasing the surfactant volume fraction Φ_I_. Results also show that, at a given Φ_I_ value, the percentage of AO in the interfacial region does not correlate with the length of the alkyl chain grafted to the antioxidants (that is, does not correlate with their hydrophobicity).

Figure 7 shows the distribution of the AOs between the aqueous, interfacial, and oil regions of the emulsions. Overall, results show that all AOs are transferred to the interfacial region, independently of the hydrophobicity, so that more than 90% of the total amount of AOs are located in this region when Φ_I_ = 0.04. As expected, a concomitant decrease in the fraction of AOs in the oil and aqueous regions is detected.

### 4.3. Effective Concentrations of AOs in the Aqueous, Interfacial, and Oil Regions of Emulsions

Chemical kinetics shows that, under the same experimental conditions, the rate of a reaction depends on the rate constant of the reaction and on the concentrations of the reactants at the reaction site. Because the inhibition reaction is competitive with the lipid oxidation reaction, the net balance of both reactions results in the relative efficiency of the antioxidant: the higher the rate of the inhibition reaction, the more efficient it is, Equation (9). This is the main purpose (and effect) of adding effective antioxidants to lipid-based emulsions.

Hence, to interpret quantitatively the experimental results, it is necessary to determine the “real” or effective concentrations of the antioxidants at the reaction site, because the “real” concentration in each region (number of moles per liter of the particular region) is different from that in the others because of the partitioning and because of the different volumes in each region and it is different from the stoichiometric one (moles of antioxidant per liter of emulsion).

In principle, we can consider that the inhibition reaction may take place simultaneously in the oil, interfacial, and aqueous regions, and thus we determined the actual concentrations in each region, which can be easily calculated from the distribution data in Figure 7 by employing Equations (24)–(26).

(24) $${(\mathrm{AO}}_{W})=\frac{{[\mathrm{AO}}_{T}{](\%\mathrm{AO}}_{W})}{\Phi_{W}}$$

(25) $${(\mathrm{AO}}_{I})=\frac{{[\mathrm{AO}}_{T}{](\%\mathrm{AO}}_{I})}{\Phi_{I}}$$

(26) $${(\mathrm{AO}}_{O})=\frac{{[\mathrm{AO}}_{T}{](\%\mathrm{AO}}_{O})}{\Phi_{O}}$$

**Figure 8:**
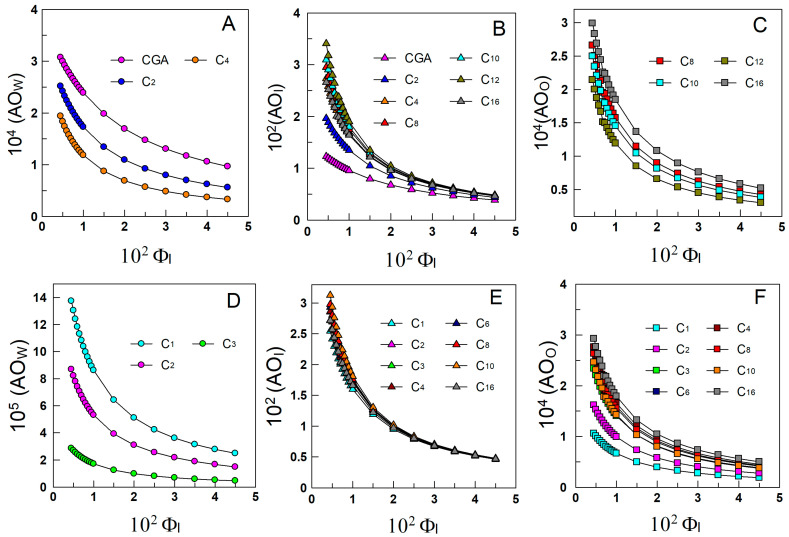
Effective concentrations (mol/L) of CGA (**upper**) and PCA (**lower**) antioxidants in the aqueous (**A**,**D**), interfacial (**B**,**E**), and oil (**C**,**F**) regions of 4:6 olive oil-in-water emulsions. [AO_T_] = 2.4 × 10^−4^ M.

Figure 8A–F displays the variations in the “real” concentration of antioxidants in the aqueous, interfacial, and oil regions of the emulsions with the number of C atoms in their alkyl chain.

Results in Figure 8 show that the effective concentration of the AOs in the aqueous (Figure 8A,D) and in the oil (Figure 8C,F) regions is much smaller than the stoichiometric one at any surfactant concentration, but that in the interfacial region is 30–300 times higher. Results also show that, upon increasing the surfactant volume fraction, antioxidants are effectively diluted in all regions. This is a consequence of competitive factors that work in opposite directions, as shown in Equation (25). On one side, antioxidants are being transferred to the interfacial region upon increasing Φ_I_, and hence the number of moles of antioxidants in the aqueous and oil regions (i.e., their percentage) decreases, hence decreasing their effective concentration (moles per liter of the particular region), and concomitantly, the number of moles of AO in the interfacial region increases. However, the extent of this increase is not compensated by the extent of the increase in the interfacial volume as a consequence of increasing Φ_I_, so that the ratio %AO_I_/Φ_I_ decreases upon increasing Φ_I,_ and hence the antioxidants are effectively diluted as they occur (for different reasons) in the aqueous and oil regions.

Results in Figure 8 also show that the effects of the length of the alkyl chain on the interfacial molarities are more significant at low Φ_I_ than at high Φ_I_, Figure 8B,E, because at high Φ_I_ most of the AOs are already located in the interfacial region.

### 4.4. Structure-Reactivity Relationships: Role of Hydrophobicity

**Figure 9:**
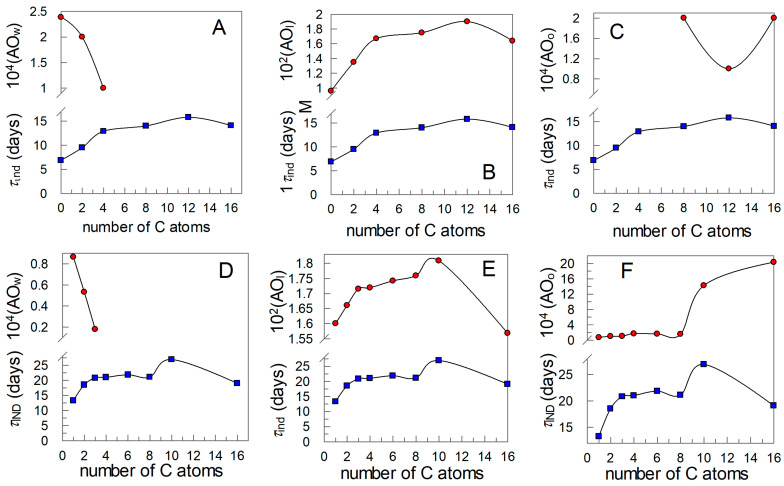
Relationships between the effective concentration of antioxidants in the aqueous, interfacial, and oil regions, the induction times, and the number of C atoms in the alkyl chain of the CGA (**A**–**C**) and PCA (**D**–**F**) derivatives, [AO_T_] = 2.4 × 10^−4^ M.

In attempting to obtain physical evidence on which region of the emulsion is mainly taking place the inhibition reaction and to acquire a better feeling on how the hydrophobicity of the antioxidant and the oil to water ratio (*o*/*w*) employed in the preparation of the emulsion affects the interfacial concentrations, we plotted, in the same graph, the variations of the effective concentrations ((AO_W_), (AO_I_), and (AO_O_)) at the low surfactant volume fraction employed (Φ_I_ = 0.01) and the variation of the induction times *τ*_ind_ against the number of C atoms in their alkyl chain, Figure 9A–F.

As shown, plots for the variations of *τ*_ind_ and (AO_I_) parallel each other but not those between *τ*_ind_ and (AO_W_) or (AO_O_). Because Equation (11) shows us that the rate of the inhibition reaction is the sum of those in the aqueous, interfacial, and oil regions, one would expect the concentration of lipid peroxides in the aqueous region, if any, to be negligible. Figure 8A,D also show that the concentration of antioxidants in the aqueous region is much lower than in the stoichiometric region and negligible for antioxidants with alkyl chains longer than 4 C atoms. Hence, the contribution of the rate in the aqueous region to the overall inhibition reaction rate should be negligible.

Figure 8C,F shows that the effective concentrations of antioxidants in the oil region are similar to the stoichiometric ones. However, peroxyl radicals that can be easily formed in the oil region because of the high concentration of unsaturated lipids in that region are expected to diffuse to more polar regions (i.e., the interfacial region) because their polarity is much higher than that of the parent lipid, making the effective concentration of peroxyl radicals in the oil region (LOO^•^_O_) to be low. Hence, one might also expect that the contribution of the rate in the oil region to the overall inhibition reaction rate is not important.

Thus, the rate of the inhibition reaction in the oil region should be much smaller than that in the interfacial region, not making a significant contribution to the rate of the overall reaction, and the overall rate of the inhibition reaction is given by Equation (27).

(27) $$r_{inh}\approx r_{inh(I)}=nk_{\mathrm{inh}(I)}{(\mathrm{LOO}}_{I}^{•}{)(\mathrm{ArOH}}_{I})$$

The finding that only the effective interfacial concentration correlates with the relative efficiency of the antioxidants, Figure 9B,E provides strong and quantitative evidence supporting the idea that the main contribution to the rate of the inhibition reaction comes from the rate in the interfacial region, i.e., the site for the reaction between peroxyl radicals and AOs in a multiphasic system is the interfacial region, as it was usually assumed in the past [12,68,69,70].

**Figure 10:**
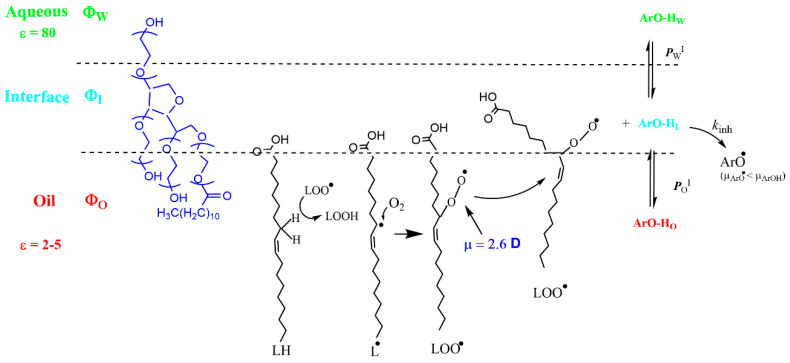
Two-dimensional pictorial representation of an emulsion droplet showing the aqueous, interfacial, and oil domains, the distribution of an antioxidant between the three regions, and the fate of a fatty acid molecule undergoing oxidation before reacting with an antioxidant. The values for the dielectric constants of the oil and aqueous regions and of the dipole moment of the formed peroxyl radical were taken from literature and are indicated to envisage the motion of the peroxyl radical towards the interfacial region. The positions and orientations of the molecules are time-averaged and are shown for illustrative purposes. Reproduced from Ref. [11], available under the Creative Commons CC-BY-NC-ND license.

Thus, the results in Figure 9 suggest a scenario close to that depicted in Figure 10, where the following features are remarkable: (I) the time-average location of ground-state antioxidants of moderate hydrophobicity is the interfacial region, and (II) the dipole moment of a PUFA molecule and that of the corresponding radical LOO^•^, which makes it diffuse to the interfacial region, reacting with the antioxidants located there.

What makes antioxidants efficient in oil-in-water emulsions?

Efficient antioxidants are those who have: (1) high reactivity (i.e., high *k*_inh_) values such that *k*_inh_ >> *k*_p_; (2) the effective concentration of the antioxidant in the interfacial region is high enough to ensure that r_inh_/r_p_ >> 1. In addition to these properties, it is also desirable that the efficient antioxidant must also be chemically stable and should not behave as a chain-transfer agent, that is, the product(s) derived from its reaction with the peroxyl radicals should not initiate new chain reactions nor contribute to carrying them on [1,10]. Since peroxyl radicals propagate peroxidation, most commonly by H-atom transfer (HAT) [10], it would be desirable to use antioxidants with a relatively weak ArO-H bond, and the bond dissociation energy generally correlates with *k*_inh_ values [10,21].

The competition between antioxidants and lipids for peroxyl (chain-carrying) radicals means that their reactivity can be readily determined based on the progress of the inhibited peroxidation reaction. Literature reports demonstrate that the kinetics of peroxidation follow the same rate laws in homogeneous and micellar solutions. However, it is necessary to consider the corresponding rate constants at the various reaction sites and the partitioning of reactants between regions. Thus, to properly describe the oxidation reaction in emulsions, it is necessary to take into account the partitioning of the reactants between the various regions of the emulsion [27,35,36,37]. However, as shown before, the main reaction site is the interfacial region, and therefore the effective concentration in that region needs to be considered. If chain termination arises from reactions of antioxidants (and the radicals derived therefrom), Equations (28) and (29) hold.

(28) $$\frac{r_{inh}}{r_{p}}=\frac{k_{inh}{(\mathrm{AO}}_{I})}{k_{p}(\mathrm{LH})}\approx A{(\mathrm{AO}}_{I})$$

(29) $$r_{i}=\frac{n\left({\mathrm{AO}}_{I}\right)}{\tau_{ind}}$$

**Figure 11:**
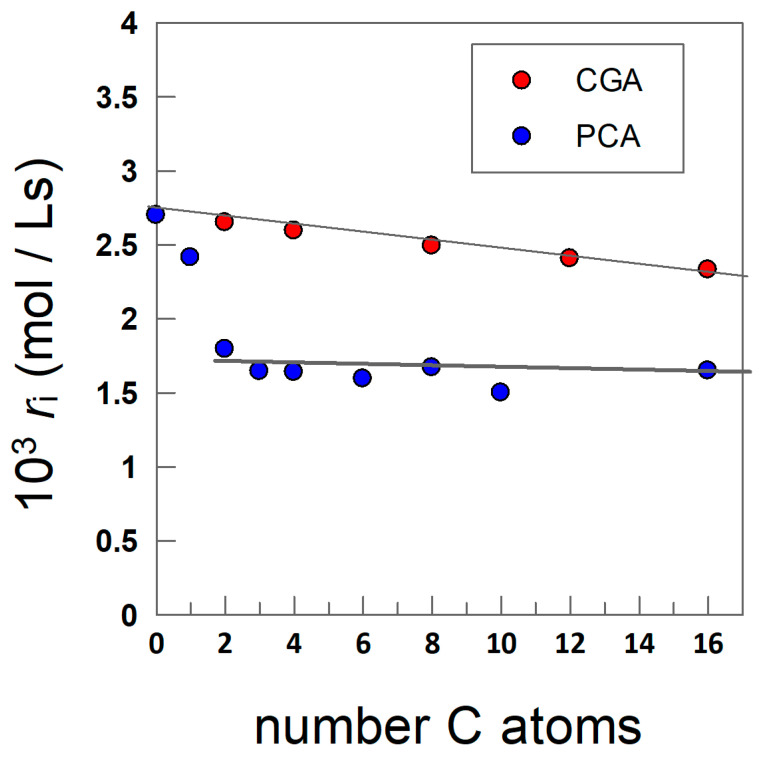
Relationships between the rate of initiation of peroxidation *r*_i_, calculated by employing Equation (29) (n = 2), and the number of C atoms in the alkyl chain of the antioxidants., The following linear relationships were obtained by fitting the experimental data. CGA, *r*_i_ = (2.7 ± 0.1) × 10^−3^ − (1.8 ± 0.3) × 10^−5^ n_C_, PCA (C2–C16), *r*_i_ = (1.7 ± 0.1) × 10^−3^ − (1 ± 1) × 10^−5^ n_C_.

If the assumption is that the interfacial region is the main reaction site for the inhibition reaction, then, according to Equation (29), the *r*_i_ values for the same homologous antioxidants should be approximately constant and independent of the length of the alkyl chain of the antioxidant because all emulsions containing the antioxidants were allowed to oxidize spontaneously under the same experimental conditions. Figure 11 shows that this prediction is essentially fulfilled for the CGA derivatives, but some deviation was found for AOs derived from PCA (C0–C2).

Unfortunately, with the current data available, we cannot offer a plausible explanation for why the *r*_i_ values for PCA and methyl protocatechuate are not constant. Calculations of the initiation rate *r*_i_ were performed under the assumption that the stoichiometric factor *n* (Equation (29)) is constant and equal to n = 2, as commonly found for most phenolic antioxidants [27,31,71]. This value is a measure of the amount of peroxyl radicals that are inactivated by a single antioxidant molecule [27]. A value of n = 2 means that one molecule of antioxidant is able to inactivate two peroxyl radicals (Equations (5) and (6)), but higher and lower fractional n values have been reported for different chain-braking antioxidants.

The constancy of the *r*_i_ values for the most hydrophobic CGA and PCA derivatives, with slopes of (1.8 ± 0.3) × 10^−5^ (CGA) and (1 ± 1) × 10^−5^ PCA, provides strong support to the idea that the efficiency of antioxidants in emulsions is controlled by both their intrinsic reactivity (which does not depend on the length of the alkyl chain) and on the effective concentrations of the antioxidants in the interfacial region, Equations (25) and (27), which in turn depend on the hydrophobicity of the antioxidant and on the surfactant concentration employed in the preparation of the emulsion. Furthermore, the constancy of the *r*_i_ values for the CGA is also consistent with the approximately constant stoichiometric numbers, expressed as moles of DPPH^•^/mol antioxidant, found by López-Giraldo et al. [72] for a series of hydrophobic CGA derivatives.

Attempts to find in the literature reports on values of the stoichiometric number n for hydrophobic derivatives of common antioxidants with lipid radicals L^•^, were not successful, and, apparently, such values have not been published so far. Similar results were found when attempting to search for relationships between n values and the nature and position of substituents on the aromatic rings of phenolic antioxidants. We, however, found n values in works where the commercially available 2,2-diphenyl-1-(2,4,6-trinitrophenyl) hydrazyl radical (DPPH^•^) was employed as a model radical. Values for the stoichiometric factors of protocatechuic acid are much more scarce than those for CGA. Villaño et al. [73] reported values of n = 2.8 (DPPH^•^ assay), while Angeli et al. [74] reported values of n = 2.1–2.5 employing the same assay. We also obtained similar values in previous works [61,62,75].

Saito et al. [76] investigated the effects of solvent on the DPPH^•^ radical scavenging ability of several protocatechuic esters. They concluded that in aprotic apolar solvents (acetone), the protocatechuic esters as well as protocatechuic acid scavenged 2.0–2.2 equivalents of the radical, but such a number increased upon increasing the polarity of the medium, the extent of the increase depending on the nature of the solvent (they reported a value of 2.8 in 1-propanol). Hence, the higher *r*_i_ values observed for the most hydrophilic PCA derivatives (C0–C2) could be eventually interpreted in terms of a change in the polarity of the region where they are sampling. In the present case, the hydrophilic PCA antioxidants may be located closer to the aqueous region and in higher concentrations compared to the most hydrophobic PCA derivatives, which are located closer to the apolar oil region because of the longer alkyl chains.

Yamamura et al. [77] compared the efficiencies of heterocyclic chain-breaking phenolic antioxidants, finding that methyl groups located in a bridging methylene C atom increase the n values compared with an ethyl group. However, the same authors report that bulky substituents such as isopropyl or phenyl groups on the bridging methylene group reduce the n value compared with the unsubstituted parent compound [77]. Hence, even though not likely, the variation in the *r*_i_ values observed between the parent PCA and the ethyl derivative (~40%) could also be interpreted in terms of a change in the stoichiometric factor n that may be affected by the addition of alkyl derivatives on going from PCA to methyl protocatechuate. Another possibility that may also be necessary to consider is the effect of solvent polarity on the reaction mechanisms. Litwinienko and Ingold [23,78] reported that polar and apolar solvents may activate different mechanisms (SPLET or HAT) and even different reaction sites (enolic or phenolic OHs). So, it may also happen that the mechanism for PCA may be slightly different from that of methyl and ethyl protocatechuates because of the different environments where they are sampling, but that such a change is not so important for CGA derivatives.

Since no definitive conclusions can be drawn to explain this apparently anomalous decrease in the *r*_i_ values for PCA, and in spite of the fact that the variation in the *r*_i_ values between the parent PCAA and the ethyl derivative is not large, ~40%, it may be worthwhile to further investigate the phenomenon. New experiments employing these and other similar antioxidants are in due course and will be part of future reports.

## 5. Conclusions

The constancy in the rate of initiation provides strong experimental evidence for a direct relationship between interfacial concentrations and antioxidant efficiencies, providing physical evidence that the effective concentration of antioxidants in the interfacial region is one of the main parameters controlling their efficiency. The results shown here also suggest that to boost antioxidant effectiveness, antioxidants with an appropriate hydrophobicity must be chosen. For example, the interfacial molarity of the C8 derivative is 75 times higher than its stoichiometric concentration ([AO_T_] = 2.4 × 10^−4^ M) when Φ_I_ = 0.005, decreasing ~6-fold upon increasing Φ_I_ to 0.04, Figure 9. More hydrophobic antioxidants, such as the C16 derivative, are less effective because they are transferred to the oil region, decreasing their effective concentration in the interfacial region.

The importance of the interfacial region in lipid peroxidation reactions has been previously highlighted by different researchers, and attempts were, and still are, made to obtain as much information as possible on the properties of this highly anisotropic region [36,37,79]. Difficulties, however, arise because of the physical impossibility of separating the interfacial region from the aqueous or oil ones without disrupting the existing equilibria, meaning that the determination of any physical parameter to characterize the region needs to be achieved in the intact emulsions.

## Data Availability

Data is contained within the article.
